# Supplementary material for: A tunable autonomous RNA-fueled micro-engine
Source: Nat Commun. 2026 Feb 25;17:3164. doi: 10.1038/s41467-026-69521-x (PMC13046739; doi:10.1038/s41467-026-69521-x)
Supplement: Supplementary file 1 — Supplementary Information [file 41467_2026_69521_MOESM1_ESM.pdf]

Supplementary Information for

A Tunable Autonomous RNA-Fueled Micro-Engine

Kun Wang*, Wenjun Chen, Buming Guo, Qiuyan Huang, Guolong Zhu, Heng Ni, Lei Zhang, Melanie Perez, Ruojie Sha, Nadrian C. Seeman and Paul M. Chaikin*

Corresponding author: Email: kunwangneu@gmail.com (K.W.); chaikin@nyu.edu (P.M.C)

The PDF file includes:

Materials
Supplementary Text
Supplementary Figure 1 to 13
Supplementary Table 1 to 4

Materials

Reagents and materials

DNA oligonucleotides and RNA were synthesized by Integrated DNA Technologies (IDT, USA) and used as received unless otherwise noted. RNA (Supplementary Table 1) were obtained from IDT with RNase-Free HPLC purification (IDT, RNase-Free HPLC grade). DNA Oligonucleotides listed in Supplementary Table 2-4 and Supplementary Data 2-5 were PAGE-purified (IDT, PAGE grade). The single-stranded M13mp18 scaffold was purchased from Tilibit Nano systems GmbH, type p7249. E. coli RNase H was from New England Biolabs (NEB, USA; Cat. M0297L). Streptavidin (Cat. 189730) and biotin-labeled bovine serum albumin (biotin-BSA) (Cat. A8549) were from Sigma-Aldrich (USA). Polystyrene microspheres (500 nm diameter) were obtained from Bangs Laboratories (USA, Cat. PS03N). DNA ladder was from Thermo Fisher Scientific (USA; Cat. SM1213). Unless specified otherwise, all other reagents were purchased from Sigma-Aldrich and used without further purification.

Supplementary Text

1. Dwell-time modeling and model selection

We analyzed n dwell times t_i measured in seconds. A right-censoring indicator $c_i \in \{0,1\}$ equals 1 when the event was unobserved at the end of recording and equals 0 otherwise. The survival function is $S(t)$ and the probability density is $f(t)$. Two parametric survivals were considered. The single-exponential survival is

$$S_1(t) = \exp\left(-\frac{t}{\tau}\right), \tag{1}$$

with density

$$f_1(t) = \tau^{-1} \exp\left(-\frac{t}{\tau}\right) \tag{2}$$

and time constant τ.

The two-component survival is

$$S_2(t) = w \, \exp\left(-\frac{t}{\tau_f}\right) + (1-w) \, \exp\left(-\frac{t}{\tau_s}\right), \tag{3}$$

with density

$$f_2(t) = w \, \tau_f^{-1} \exp\left(-\frac{t}{\tau_f}\right) + (1-w) \, \tau_s^{-1} \exp\left(-\frac{t}{\tau_s}\right), \tag{4}$$

where $w \in (0,1)$ is the mixing weight of the fast subpopulation, τ_f is the fast time constant, and τ_s is the slow time constant.

Parameter estimation used maximum likelihood with censoring. The parameter vector is θ. The log-likelihood is

$$\log L(\theta) = \textstyle\sum_{i:c_i=0} \log f(\, t_i \mid \theta \,) + \sum_{i:\, c_i=1} \log S(\, t_i \mid \theta \,). \tag{5}$$

Feasibility was enforced by reparameterization. The weight used

$$w=\sigma(a) \tag{6}$$

with

$$\sigma(a) = \frac{1}{1+\mathrm{e}^{-a}}. \tag{7}$$

Time constants used exponential maps so that

$$\tau = \mathrm{e}^{b} \tag{8}$$

for the single-exponential model and

$$\tau_f = \mathrm{e}^{b_f} \tag{9}$$

and

$$\tau_s = \mathrm{e}^{b_s} \tag{10}$$

for the two-component model, where a, b, b_f, *and* b_s are unconstrained real variables in the optimizer. Multi-start numerical optimization was applied to reduce local minima. Fits used the raw dwell times without binning.

Model comparison used the Akaike information criterion and the Bayesian information criterion. The formulas are

$$\mathrm{AIC} = 2k - 2\log L(\hat{\theta}), \mathrm{BIC} = k\ln n - 2\log L(\hat{\theta}), \tag{11}$$

where k is the number of free parameters and $\hat{\theta}$ is the maximum-likelihood estimate. For the single-exponential model k=1 with parameter τ. For the two-component model k=3 with parameters w, τ_f, and τ_s. We report

$$\Delta\mathrm{AIC} = \mathrm{AIC}_{\mathrm{single}} - \mathrm{AIC}_{\mathrm{double}} \tag{12}$$

and

$$\Delta\mathrm{BIC} = \mathrm{BIC}_{\mathrm{single}} - \mathrm{BIC}_{\mathrm{double}}. \tag{13}$$

Positive values favor the two-component model.

Statistical evidence for improvement was assessed by a likelihood-ratio test with a parametric bootstrap under the null model S_1. The observed statistic is

$$\mathrm{LR}_{\mathrm{obs}} = 2\left[\log L_{\mathrm{double}}(\hat{\theta}_{\mathrm{double}}) - \log L_{\mathrm{single}}(\hat{\theta}_{\mathrm{single}})\right]. \tag{14}$$

We generated B bootstrap datasets from the single-exponential model using $\hat{\theta}_{\mathrm{single}}$ and preserved the sample size n and the censoring pattern $\{c_i\}$. Each bootstrap dataset was refit by both models to compute LR_b. The p-value estimate used the add-one correction

$$\hat{p} = \frac{\#\{\mathrm{LR}_b \geq \mathrm{LR}_{\mathrm{obs}}\}+1}{B+1}, \tag{15}$$

where B is the number of bootstrap replicates.

For cross-condition comparisons we summarize kinetics by the mean dwell time

$$\langle t \rangle = w\,\tau_f + (1-w)\,\tau_s, \tag{16}$$

which equals the first moment of the two-component survival. Uncertainty for $\langle t \rangle$ was obtained by nonparametric bootstrap resampling of $\{t_i\}$ with replacement followed by refitting. Results are reported as the bootstrap standard error.

2. Simulation of the τ_C of the RNA-fueled engine

To quantitatively describe the kinetics of RNA cleavage by RNase H, we constructed a simple three-state model based on classical enzyme-substrate interactions (Supplementary Figure 8). In this model, the substrate consists of a DNA/RNA duplex (denoted as DNA~RNA), which binds reversibly with the enzyme (RNase H) to form a complex (DNA~RNA~Enzyme). This complex then undergoes catalysis to release cleaved DNA (product) and regenerate the free enzyme. The associated system of ordinary differential equations (ODEs) describing the time-dependent evolution of each species is given by:

$$\frac{d[DNA\sim RNA]}{dt} = -k_a[DNA\sim RNA][Enzyme] + k_d[DNA\sim RNA\sim Enzyme] \tag{17}$$

$$\frac{d[DNA\sim RNA\sim Enzyme]}{dt} = k_a[DNA\sim RNA][Enzyme] - (k_{cat} + k_d)[DNA\sim RNA\sim Enzyme] \tag{18}$$

$$\frac{d[DNA]}{dt} = k_{cat}[DNA\sim RNA\sim Enzyme] \tag{19}$$

This minimal kinetic model captures both the reversible enzyme binding (association rate k_a and dissociation rate k_d) and the irreversible cleavage step characterized by the catalytic rate k_{cat}. The model allows for fitting experimental data to extract parameters such as the characteristic cleavage time τ_C.

The cumulative distribution functions (CDFs) of the folded dwell times at different enzyme concentrations were globally fitted using the ODE model described above. All datasets were simultaneously fit with shared kinetic parameters, as shown in Supplementary Figure 9. The best-fit values were: k_a : 0.4 x 10^6 $M^{-1}\cdot s^{-1}$, k_d :0.4 s^{-1}, k_{cat} :98 s^{-1}. The fitted values for k_a and k_d are on the same order of magnitude as previously reported for RNase H in similar systems[36,37]. However, the extracted k_{cat} is significantly higher than literature values, which typically report cleavage rates of ~1 s^{-1}. This discrepancy likely arises because only partial RNA cleavage is sufficient to activate the DNA engine in our system, whereas literature values typically refer to complete RNA digestion. This interpretation is also supported by native gel electrophoresis results (Supplementary Figure 4).

3. Simulation of the τ_O of the RNA-fueled engine

To investigate how the unfolded dwell time (τ_o) depends on RNA and enzyme concentrations, we developed a kinetic model that explicitly tracks all relevant intermediate states during the engine folding process. As illustrated in Supplementary Figure 10, kinetic pathway model describing the dwell time in the unfolded state of the engine. RNA binds to the DNA with rate constant k_1, forming an intermediate DNA~RNA hybrid. From this state, the system can either proceed to the folded state with rate k_{on} or revert to the initial DNA state via enzyme binding (association rate k_a, dissociation rate k_d) followed by cleavage at rate k_{cat}. A competing pathway emerges: a second RNA strand can hybridize with rate k_1, generating a misfolded DNA~2RNA complex. Rescue from the misfolded state occurs via enzyme binding and cleavage, reducing RNA occupancy and returning the system to the earlier intermediate. The associated system of ordinary differential equations (ODEs) describing the time-dependent evolution of each species is given by:

$$\frac{d[DNA]}{dt} = -k_1[RNA][DNA] + k_{cat}[DNA \sim RNA \sim Enzyme] \quad (20)$$

$$\frac{d[DNA\sim RNA]}{dt} = k_1[RNA][DNA] - k_a[Enzyme][DNA \sim RNA] + k_d[DNA \sim RNA \sim Enzyme] - k_1[RNA][DNA \sim RNA] - k_{on}[DNA \sim RNA] + k_{cat}[DNA{\sim}2RNA{\sim}Enzyme] \quad (21)$$

$$\frac{d[DNA\sim RNA\sim Enzyme]}{dt} = k_a[Enzyme][DNA \sim RNA] - k_d[DNA \sim RNA \sim Enzyme] - k_{cat}[DNA \sim RNA \sim Enzyme] \quad (22)$$

$$\frac{d[DNA\sim 2RNA]}{dt} = k_1[RNA][DNA \sim RNA] - k_a[Enzyme][DNA \sim 2RNA] + k_d[DNA \sim 2RNA \sim Enzyme] \quad (23)$$

$$\frac{d[DNA\sim 2RNA\sim Enzyme]}{dt} = k_a[Enzyme][DNA \sim 2RNA] - k_d[DNA \sim 2RNA \sim Enzyme] - k_{cat}[DNA \sim 2RNA \sim Enzyme] \quad (24)$$

$$\frac{d[Folded]}{dt} = k_{on}[DNA \sim RNA] \quad (25)$$

This model captures the essential interplay between productive and unproductive folding kinetics and highlights the enzyme's role in rescuing stalled intermediates. When we applied the model to globally fit experimental τ_o distributions across a range of RNA and enzyme concentrations, we observed generally good agreement between simulation and experiment, as shown in Supplementary Figure 11. However, in some cases, deviations were apparent.

To quantitatively evaluate the agreement between the experimental dwell-time distributions and the global kinetic model (Supplementary Figure 11), we calculated two metrics for each experimental condition: the Kolmogorov-Smirnov (K-S) statistic (D) and the Root Mean Square Error (RMSE). The K-S statistic D quantifies the maximum vertical deviation between the experimental CDF, denoted as $CDF_{exp}(t)$, and the model-predicted CDF, $CDF_{model}(t)$:

$$D = \max_{\{t\}} \left| CDF_{exp}(t) - CDF_{model}(t) \right| \quad (26)$$

The RMSE was computed to assess the overall deviation across the entire time course for each condition:

$$RMSE = \sqrt{\frac{1}{N}\sum_{i=1}^{N}\left(CDF_{\exp}(t_i) - CDF_{\mathrm{model}}(t_i)\right)^2} \quad (27)$$

Where N is the total number of experimental data points in the specific condition, and $CDF_{model}(t_i)$ is the simulated probability interpolated to the experimental time point t_i.To provide

a summary metric for the global fit quality across the entire dataset, the D_{avg} was calculated as the arithmetic mean of the individual D statistics obtained from all 11 experimental conditions:

$$D_{avg} = \frac{1}{M}\sum_{j=1}^{M} D_j \tag{28}$$

where M = 11 represents the total number of varying RNA and Enzyme concentration conditions analyzed. The calculated average K-S statistic ($D_{avg} = 0.17$) demonstrates that the global kinetic model effectively captures the dominant mechanistic features of the system across a broad range of experimental conditions. Given that the model employs a single set of rate constants to describe the dwell-time distributions across 11 different RNA and enzyme concentrations, an average maximum deviation of approximately 0.17 represents a satisfactory level of agreement. We suspect that a major contributing factor for the deviation is the use of running average smoothing in the experimental data analysis, which may obscure short-lived transition events and thus underestimate fast dynamics. Additionally, discrepancies may reflect further kinetic complexity in the real system—such as asynchronous handle binding or hidden conformational substates—not captured by our idealized model. Despite these limitations, the model successfully recapitulates the overall trend: τ_o decreases with increasing RNA concentration and enzyme activity, consistent with the hypothesis that both faster hybridization and more efficient misfolding clearance accelerate the folding process.

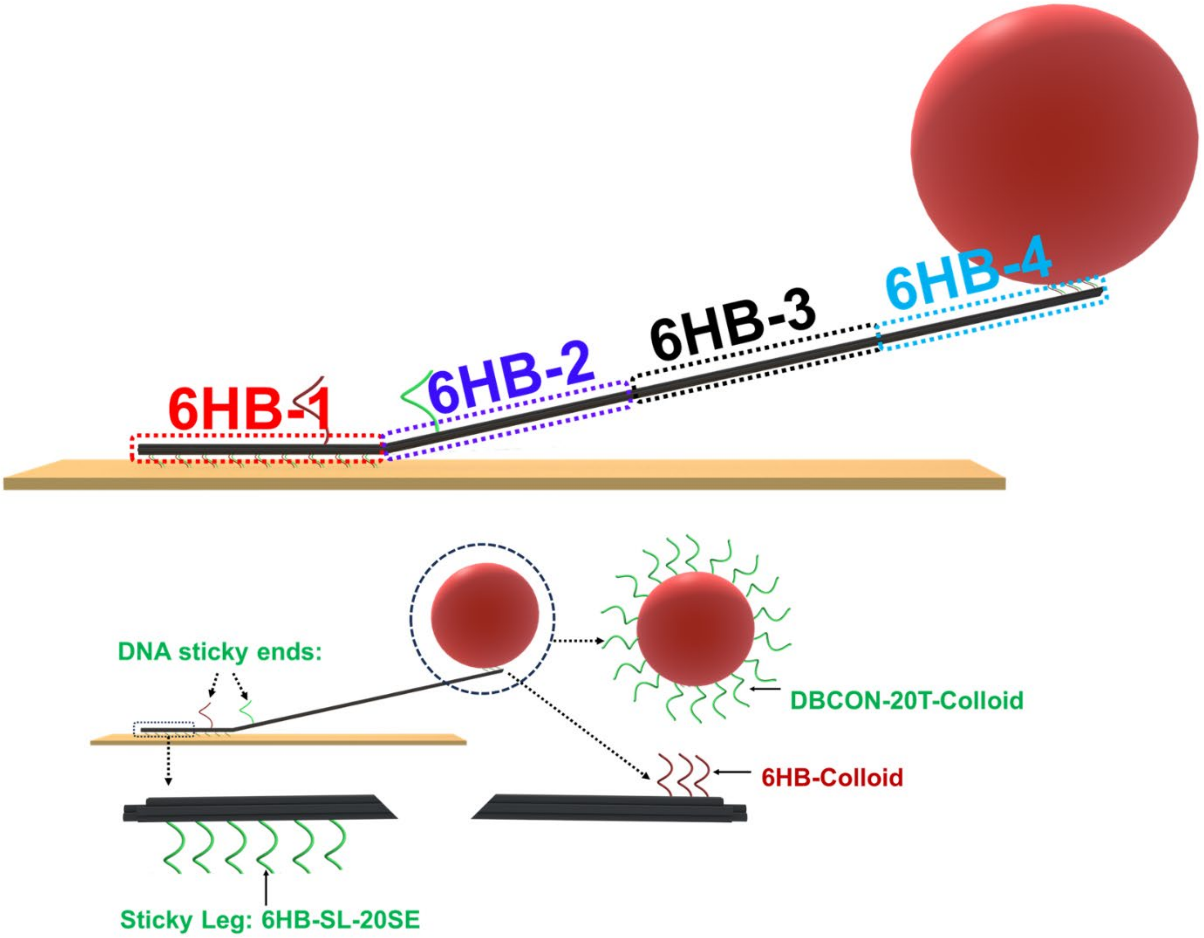

Supplementary Figure 1. Detailed strand design of the RNA-fueled engine. Schematic illustration depicting the DNA strands used in RNA-fueled engine.

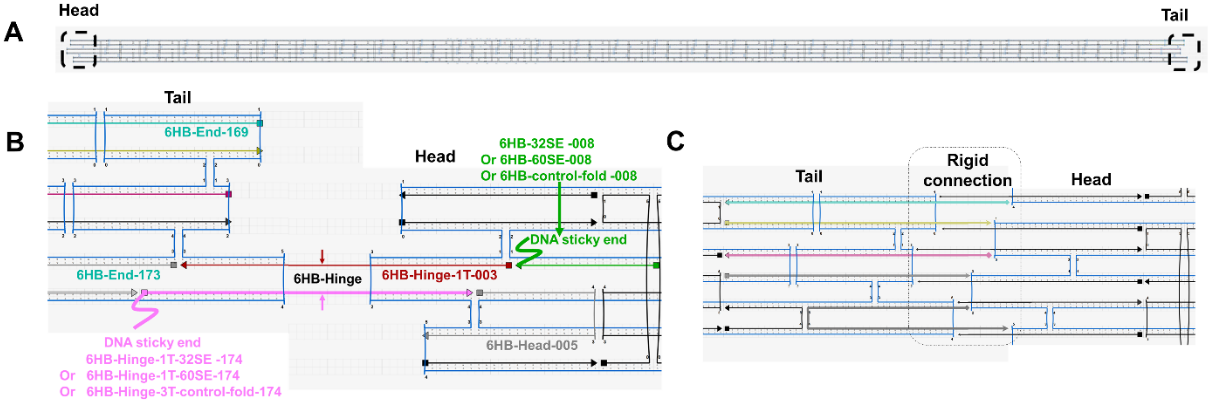

Supplementary Figure 2. Structural details of the 6-helix bundle, hinge, and rigid connections. Schematic illustration depicting the 6HB (A), the hinge (B) and rigid connection structure (C) used in RNA-fueled engine. Labels in (B) match the sequence names in Supplementary Table 3 (sticky ends and hinge springs). 6HB-Hinge-1T-32SE-174 and 6HB-32SE-008 were used for all experiments. 6HB-Hinge-1T-60SE-174, 6HB-60SE-008, 6HB-Hinge-3T-Control-Fold-174, and 6HB-Control-Fold-008 were used only in Supplementary Figure 4 ("Folding behavior of 6HB dimers assembled with RNA linkers of varying lengths").

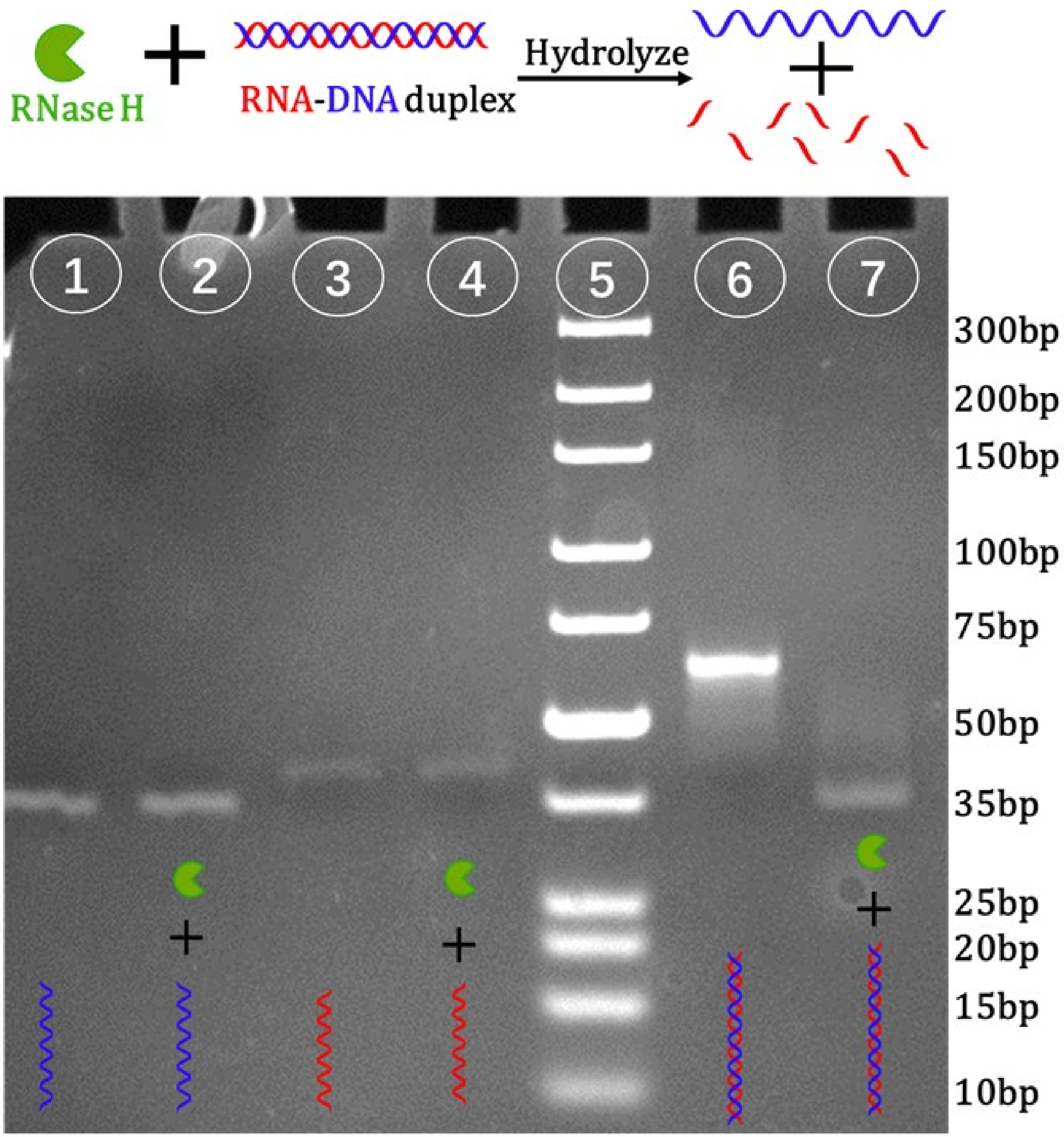

Supplementary Figure 3. Verification of RNase H activity and substrate specificity. RNase H activity assay evaluated using a 10% native polyacrylamide gel electrophoresis. Lane 1: single-stranded DNA (ssDNA) only. Lane 2: ssDNA with RNase H. Lane 3: single-stranded RNA (ssRNA) only. Lane 4: ssRNA with RNase H. Lane 5: ultra-low range DNA ladder. Lane 6: DNA/RNA duplex. Lane 7: DNA/RNA duplex treated with RNase H. The cleavage of RNA in the DNA/RNA duplex (Lane 7) indicates RNase H activity, while the lack of digestion in lanes 2 and 4 confirms its substrate specificity. Uncropped gel image are provided as a Source Data file.

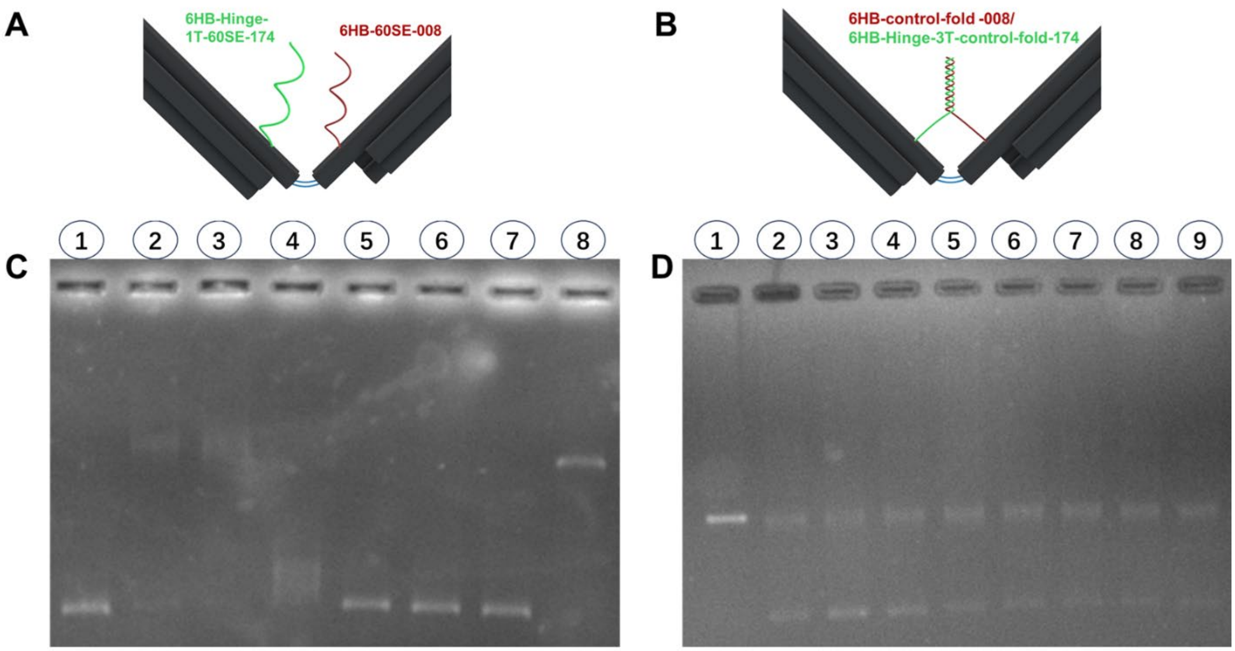

Supplementary Figure 4. Effect of RNA linker length on the folding behavior of 6HB dimers. Folding behavior of 6HB dimers assembled with RNA linkers of varying lengths, resolved by 0.7% native agarose gel. (A) Experimental design used for this assay: 6HB-Hinge-1T-60SE-174 paired with 6HB-60SE-008 so that RNA linkers of different lengths can hybridize to the same long DNA sticky ends (see Supplementary Table 3 for sticky-end and hinge-spring sequences). (B) Folded-state positive control formed by complementary DNA sticky ends only (no RNA). (C) Lanes 1–8: lane 1, no RNA linker (unfolded control); lanes 2–7, RNA linkers of 32, 28, 24, 20, 16, 12 nt; lane 8, folded-state control as in (B). (D) Lanes 1–9: lane 1, folded-state control as in (B); lanes 2–9, RNA linkers of 32, 36, 40, 44, 48, 52, 56, 58 nt. RNA linker sequences are listed in Supplementary Table 1; all sticky-end and hinge-spring sequences used here are listed in Supplementary Table 3. Uncropped gel image are provided as a Source Data file.

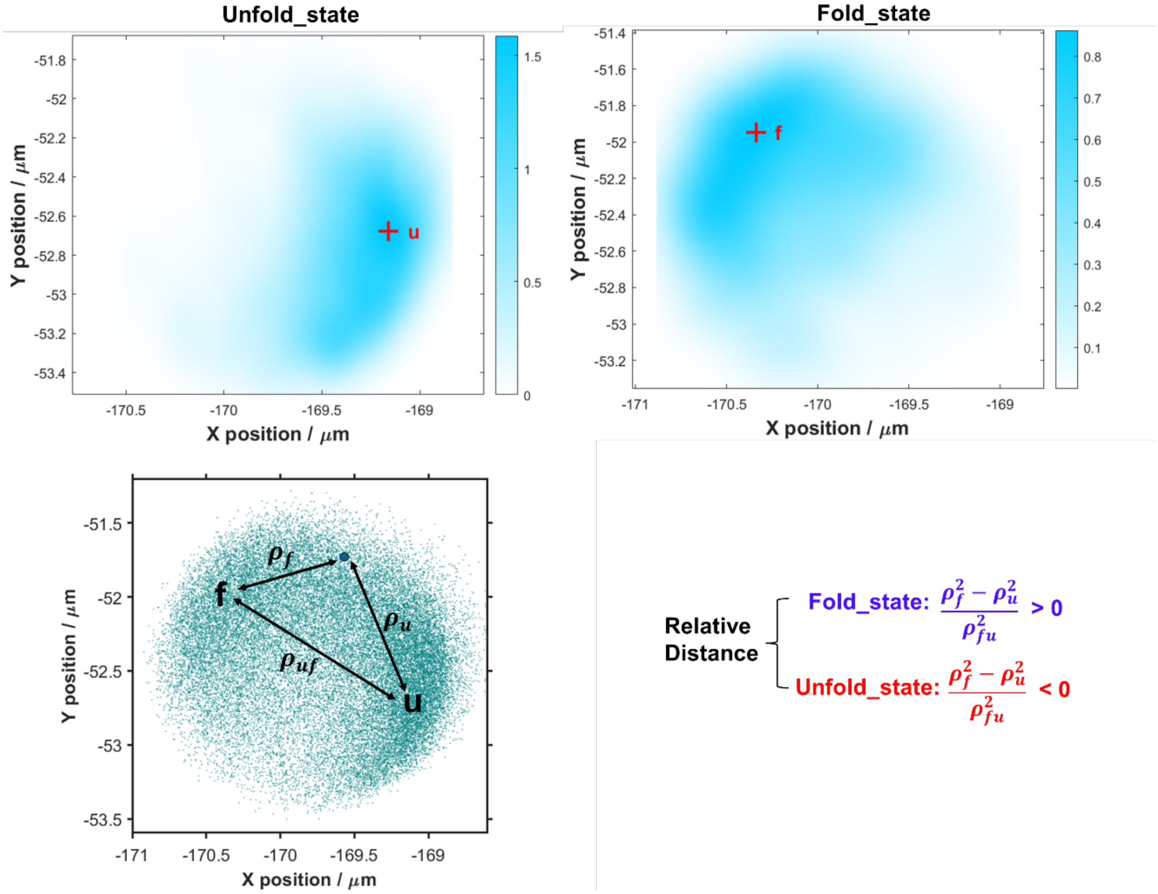

Supplementary Figure 5. Definition of the relative position metric used for state classification. First, determine the point of maximum likelihood for the unfold state (u) without RNA linker. Next, identify the point of maximum likelihood for the fold state (f) with RNA linker. Subsequently, compute the distance between the colloid's position and the u point ($\boldsymbol{\rho}_{\mathrm{u}}$) at a specific time, as well as the distance between the colloid's position and the f point ($\boldsymbol{\rho}_{\mathrm{f}}$), and the distance between f and u ($\boldsymbol{\rho}_{\mathrm{fu}}$). As shown in Fig. 2,3 and 5 in main text, determine the colloid's state by evaluating the expression $(\boldsymbol{\rho}_f^2 - \boldsymbol{\rho}_u^2)/(\ \boldsymbol{\rho}_{fu}^2)$. This quantity is negative in the unfolded state and positive in the folded state.

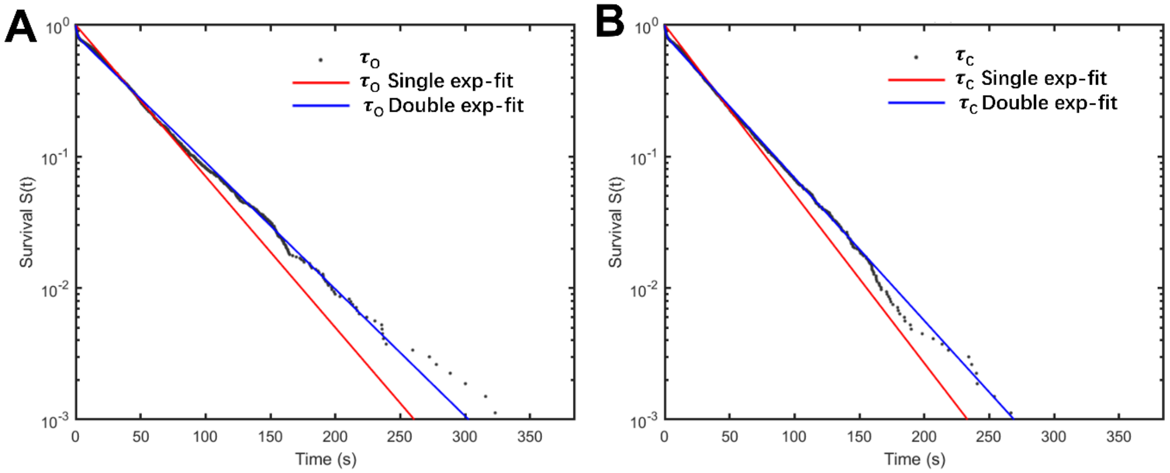

Supplementary Figure 6. Comparison of single- and double-exponential models for dwell time analysis. Black dots show the Kaplan–Meier estimate $S(t) = 1 - \mathrm{CDF}(t)$ from the raw dwell times (Data were collected at 37 °C with a frame rate of 20 FPS with both RNA (500 nM) and RNase H (80 nM)). Red lines are single-exponential maximum-likelihood fits. Blue lines are two-component survival fits

$$S(t) = w\ \exp\left(-\frac{t}{\tau_f}\right) + (1 - w)\ \exp\left(-\frac{t}{\tau_s}\right), \tag{29}$$

where $w$is the fast-component weight, $\tau_f$the fast time constant, and $\tau_s$the slow time constant. (A) Unfolded macrostate τ_O. The early-time shoulder and long tail require two time scales. Model selection supports the two-component description with ΔAIC 771.4, ΔBIC 759.7, and a bootstrap likelihood-ratio test $p < 2.5 \times 10^{-3}$ (B = 400). (B) Folded macrostate τ_C. The same trend is observed with ΔAIC 713.5, ΔBIC 701.7, and $p < 2.5 \times 10^{-3}$. Model survival curves $S_1(t)$ and $S_2(t)$ are overlaid on semilogarithmic axes for visualization only. All estimates and tests rely on the likelihood formulations at Supplementary Text 1.

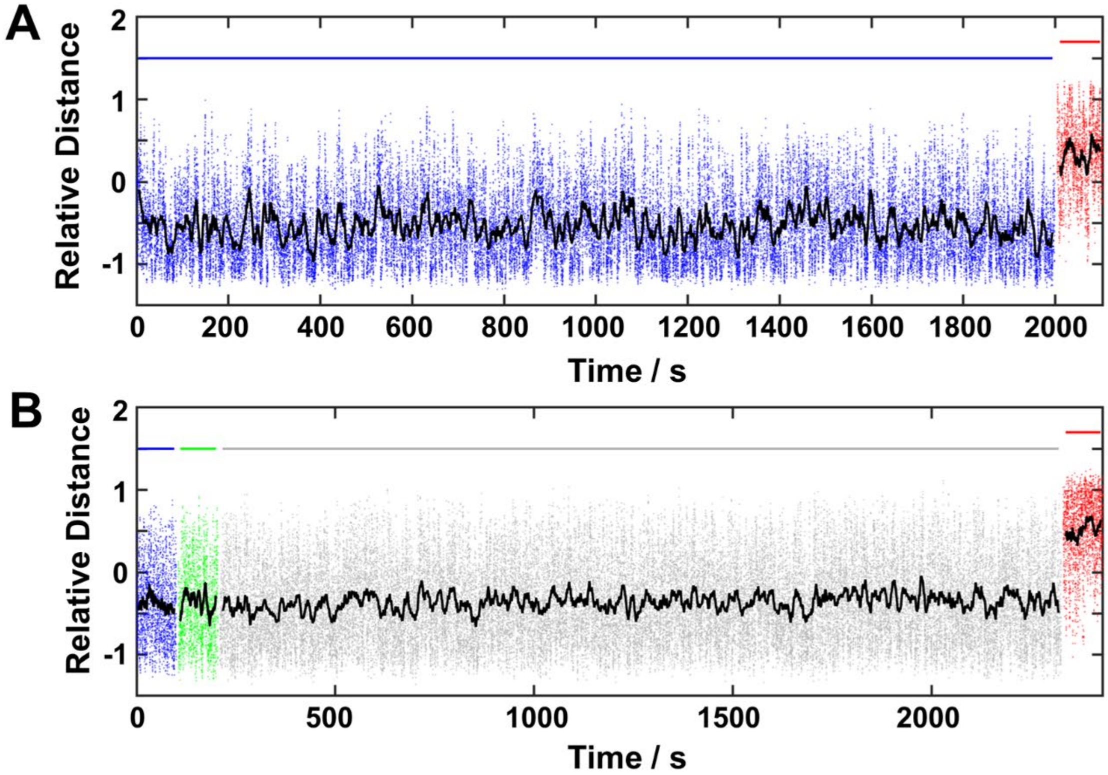

Supplementary Figure 7. Control experiments establishing state references and operation specificity. Time traces of the particle's relative distance with a 20-second running-average overlay (black). (A) Control recordings to define state references: unfolded control without RNA or RNase H (blue) and folded control with RNA linker (500 nM, red). The top binary bar shows idealized state calls obtained by position-thresholding. (B) Specificity and actuation controls: scrambled RNA linker alone (500 nM, green) and scrambled RNA + RNase H (500 nM and 55 nM, gray), alongside the unfolded (blue) and folded (red) controls. The top binary bar again reports the idealized state assignments. Data were collected at 37 °C with a frame rate of 20 FPS.

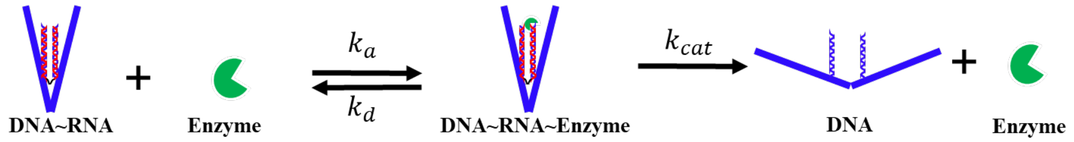

Supplementary Figure 8. Reaction scheme used to model the kinetics of RNA cleavage by RNase H. A simple three-state model was constructed based on classical enzyme–substrate interactions. In this model, the DNA/RNA duplex (DNA~RNA) serves as the substrate, which reversibly binds with RNase H to form a DNA/RNA/Enzyme complex with association and dissociation rate constants k_a and k_d, respectively. The complex then undergoes irreversible catalytic cleavage with rate constant $k$cat, resulting in the release of DNA and the regeneration of the enzyme.

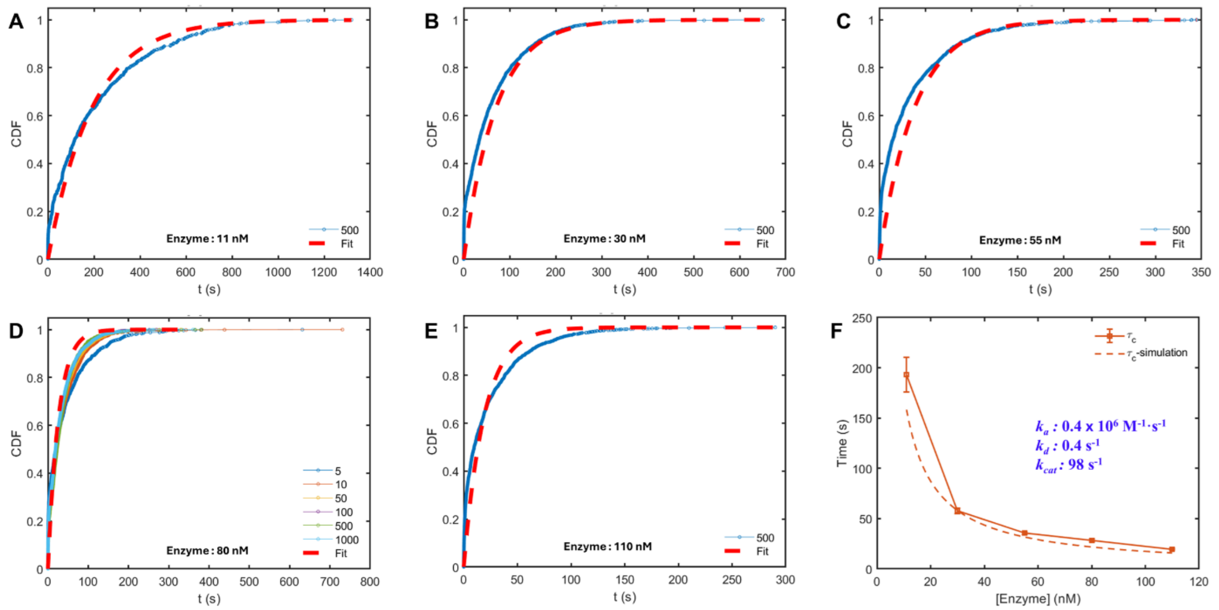

Supplementary Figure 9. Global fitting of folded-state dwell time distributions. (A-E), Cumulative distribution functions (CDFs) of the folded-state dwell times (τ_C) at various enzyme concentrations (11–110 nM). The solid blue curves represent experimental data at different RNA concentrations (indicated by line color; unit: nM), and the red dashed curves show the global fit using the model described in Supplementary Figure 8. (F) Comparison of τ_C values obtained from double-exponential fitting of the experimental data (the solid line with error bars is replotted from Figure 4L) and simulations using the best-fit kinetic parameters (dashed line). The fitted rate constants are: $k_a = 0.4 \times 10^6$ $M^{-1}s^{-1}$, $k_d = 0.4$ s^{-1}, $k_{cat} = 98$ s^{-1}.

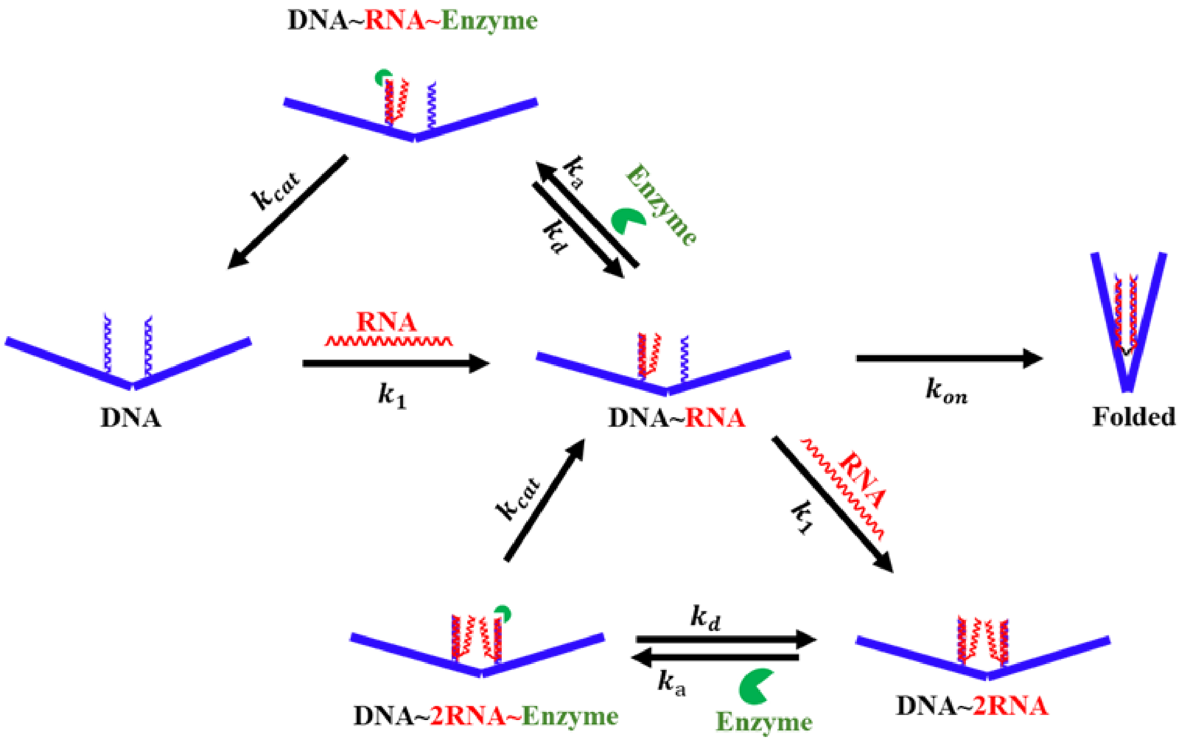

Supplementary Figure 10. Kinetic pathway model of the unfolded state. Kinetic pathway model describing the dwell time in the unfolded state of the engine.

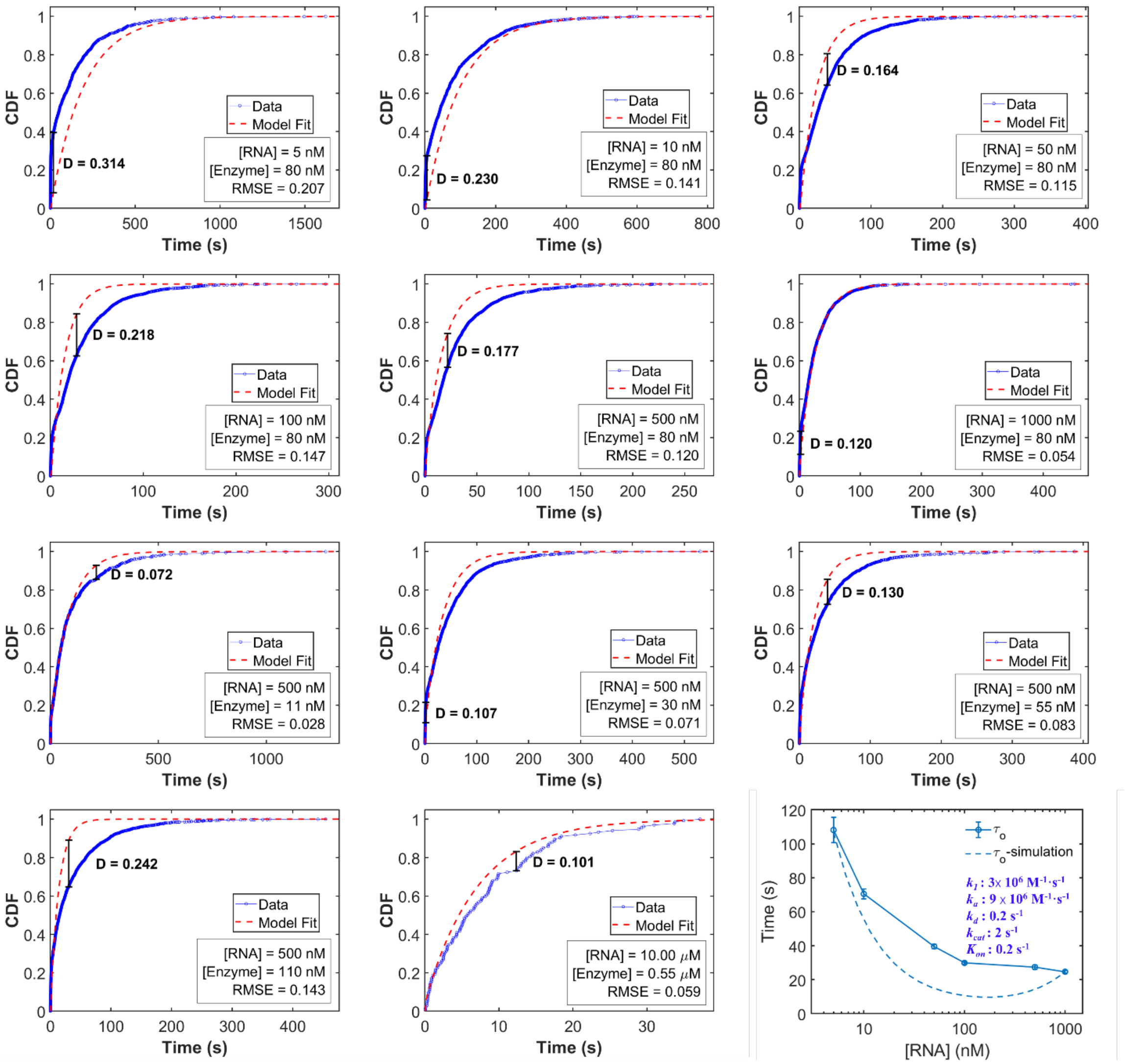

Supplementary Figure 11. Global fitting and statistical analysis of unfolded-state dwell time distributions. Cumulative distribution functions (CDFs) of the unfolded-state dwell times (τ_O) under various RNA and enzyme concentrations. Blue curves represent experimental data, and red curves show the global fits based on the kinetic model described in Supplementary Figure 10. The quality of the fit is quantified by two metrics: the Kolmogorov-Smirnov statistic (D), indicated by a vertical black line segment representing the maximum deviation between the experimental and simulated distributions, and the Root Mean Square Error (RMSE), listed in the inset with the corresponding [RNA] and [Enzyme] conditions. The analysis yielded an average K-S statistic of $D_{avg} = 0.17$. Given that the model employs a single set of rate constants to describe the dwell-time distributions across 11 different RNA and enzyme concentrations, this average maximum deviation of approximately 0.17 represents a satisfactory level of agreement. The bottom-right panel summarizes the τ_o from experiment (The solid line with error bars is replotted from Figure 4F) and simulated τ_O values (dashed line) as a function of RNA concentration (log scale).The best-fit rate constants used in the model are: $k_1 = 3 \times 10^6$ $M^{-1}s^{-1}$, $k_a = 9 \times 10^6$ $M^{-1}s^{-1}$, $k_d = 0.2$ s^{-1}, $k_{cat} = 2$ s^{-1}, $k_{on} = 0.2$ s^{-1}.

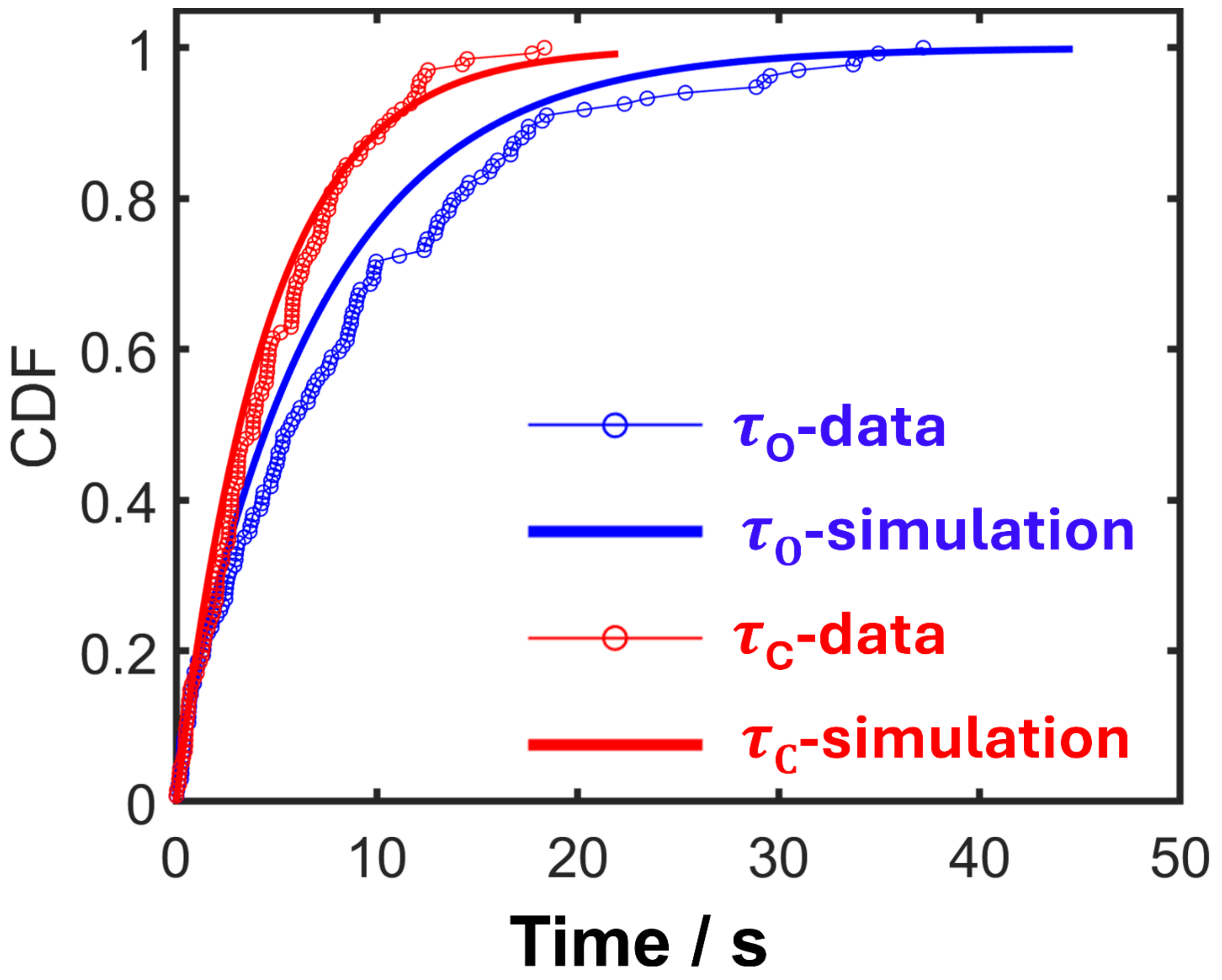

Supplementary Figure 12. Validation of the kinetic model under high-concentration conditions. Cumulative distribution functions (CDFs) of dwell times in the unfolded state (τ_O, blue) and folded state (τ_C, red), measured under high RNA and RNase H concentrations (RNA: 10 μM; RNase H: 550 nM). Open circles represent experimental data, and solid lines represent model simulations. The simulation results were generated using the kinetic model described in Supplementary Figure 8 and 10, with parameters extracted from the fits in Supplementary Figure 9 and 11. The close agreement between simulation and experimental CDFs validates the model's ability to capture both τ_O and τ_C dynamics under conditions of rapid cycling.

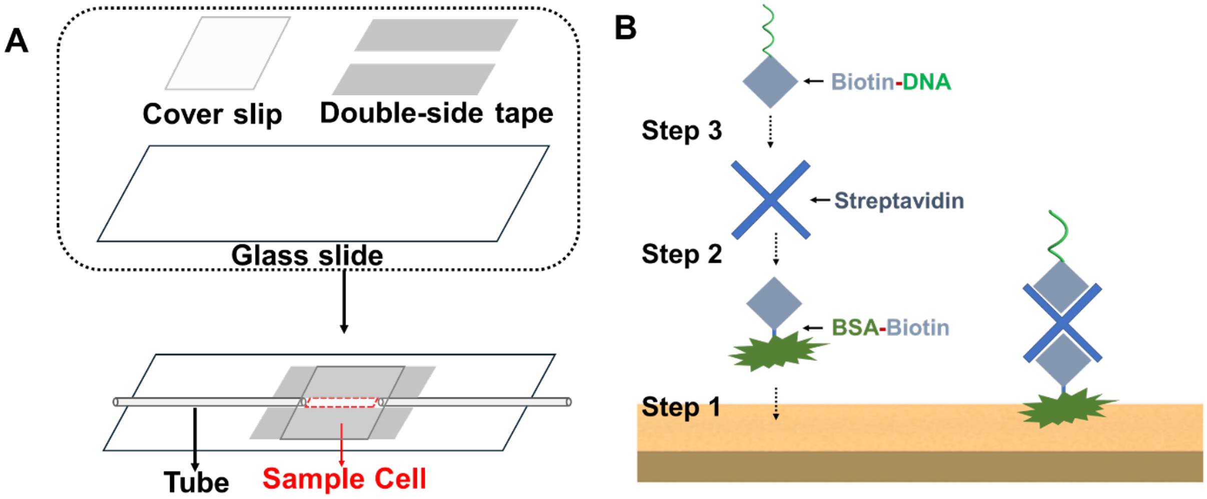

Supplementary Figure 13. Procedures for constructing and modifying the experimental chamber. (A) The process of assembling the sample cell. (B) The procedure for functionalizing the surface of the sample cell.

Supplementary Table 1.

The RNA strand sequence is designed for the RNA linker of the RNA-fueled engine.

**Name**	**Sequence**
RNA linker-12SE	rUrU rArArU rC rU rG rArGrG rArG
RNA linker-16SE	rC rUrUrU rArArU rC rU rG rArGrG rArGrU rC
RNA linker-20SE	rArArC rUrUrU rArArU rC rU rG rArGrG rArGrU rCrArU
RNA linker-24SE	rUrA rArArC rUrUrU rArArU rC rU rG rArGrG rArGrU rCrArU rUrC
RNA linker-28SE	rG rCrUrA rArArC rUrUrU rArArU rC rU rG rArGrG rArGrU rCrArU rUrCrA rC
RNA linker-32SE	rG rUrG rCrUrA rArArC rUrUrU rArArU rC rU rG rArGrG rArGrU rCrArU rUrCrA rCrC rG
RNA linker-36SE	rGrArG rUrG rCrUrA rArArC rUrUrU rArArU rC rU rG rArGrG rArGrU rCrArU rUrCrA rCrC rGrUrC
RNA linker-40SE	rArC rGrArG rUrG rCrUrA rArArC rUrUrU rArArU rC rU rG rArGrG rArGrU rCrArU rUrCrA rCrC rGrUrC rUrG
RNA linker-44SE	rG rGrArC rGrArG rUrG rCrUrA rArArC rUrUrU rArArU rC rU rG rArGrG rArGrU rCrArU rUrCrA rCrC rGrUrC rUrGrG rA
RNA linker-48SE	rUrArG rGrArC rGrArG rUrG rCrUrA rArArC rUrUrU rArArU rC rU rG rArGrG rArGrU rCrArU rUrCrA rCrC rGrUrC rUrGrG rArArC
RNA linker-52SE	rCrA rUrArG rGrArC rGrArG rUrG rCrUrA rArArC rUrUrU rArArU rC rU rG rArGrG rArGrU rCrArU rUrCrA rCrC rGrUrC rUrGrG rArArC rArA
RNA linker-56SE	rG rArCrA rUrArG rGrArC rGrArG rUrG rCrUrA rArArC rUrUrU rArArU rC rU rG rArGrG rArGrU rCrArU rUrCrA rCrC rGrUrC rUrGrG rArArC rArArU rA
RNA linker-60SE	rUrGrG rArCrA rUrArG rGrArC rGrArG rUrG rCrUrA rArArC rUrUrU rArArU rC rU rG rArGrG rArGrU rCrArU rUrCrA rCrC rGrUrC rUrGrG rArArC rArArU rArCrC
RNA-scrambled linker	rU rCrCrG rGrArC rUrArG rGrUrU rArCrC rGrCrU rU rCrArU rUrCrU rArArG rC rUrGrC

Supplementary Table 2.

The specific DNA staple strand sequence is designed for the sticky ends of the RNA-fueled engine, which is used to connect with colloids (Supplementary Figure 1). Note: The last three digits in the name represent the position of the staple strand that needs to be replaced.

**Name**	**Sequence**
6HB-colloid-front-152	ATGGCTAGTAAATGCTTAGGT TTTTTTTTTTTTTT ATGAACCTGCTGTTGCACTAG
6HB-colloid-rear-152	TGGGTTATTATCATAGGTCTG TTTTTTTTTTTTTT ATGAACCTGCTGTTGCACTAG
6HB-colloid -front-153	TTAATTTTCCCTAAGTAAATC TTTTTTTTTTTTTT ATGAACCTGCTGTTGCACTAG
6HB-colloid -rear-153	GTCGCTATAGAATAACCTTGC TTTTTTTTTTTTTT ATGAACCTGCTGTTGCACTAG
6HB-colloid -front-158	ATCGCCAATGGAAAAATTTCA TTTTTTTTTTTTTT ATGAACCTGCTGTTGCACTAG
6HB-colloid -rear-158	TTTGAATCGACAAACATCAAG TTTTTTTTTTTTTT ATGAACCTGCTGTTGCACTAG
6HB-colloid -front-159	TGATTGCTTTGAATAACAATA TTTTTTTTTTTTTT ATGAACCTGCTGTTGCACTAG
6HB-colloid -rear-159	ACGGATTTACTTTTACATCGG TTTTTTTTTTTTTT ATGAACCTGCTGTTGCACTAG
6HB-colloid -front-164	AACAGAGGTTTAACATAAAGA TTTTTTTTTTTTTT ATGAACCTGCTGTTGCACTAG
6HB-colloid -rear-164	AATTGCGCACCTACCATATCA TTTTTTTTTTTTTT ATGAACCTGCTGTTGCACTAG
6HB-colloid -front-165	TTCCTGATTATCTAAGCGGAA TTTTTTTTTTTTTT ATGAACCTGCTGTTGCACTAG
6HB-colloid -rear-165	TTATCATTAAGAAACCACCAG TTTTTTTTTTTTTT ATGAACCTGCTGTTGCACTAG

Supplementary Table 3.

The specific DNA staple strand sequence is designed for the DNA sticky ends and the hinge spring of the RNA-fueled engine (Supplementary Figure 1 and 2). Note: The last three digits in the name represent the position of the staple strand that needs to be replaced.

Name	Sequence
6HB-Hinge-1T-003	CATAAAGTGTAAAGCCTGG T TCACCTTGCTGAAC
6HB-Head-005	TGCCAAGCTTGCATGCCTAAAACGACGGCCAGTGAGCTAATTTT
6HB -front-008	ACTGCCCGAAATTGTCATGGTCATAGCTATCTAG
6HB-32SE -008	AGGATCCCCGGGTACCGAG T GAT TAA AGT TTA GCA C
6HB-End-169	TTTTCAATCAATATCTGGTCAGTTGTAATTTTAATGAGAGCCAGCAGC
6HB-End-173	TTTAGGAGCACTAACAACTAATAGAT
6HB-Hinge-1T-32SE -174	CGG TGA ATG ACT CCT C T AAATGAAAAATCTAAAGCA T GGTGCCTAATGAG
6HB-Hinge-1T-60SE-174	GGT ATT GTT CCA GAC GGT GAA TGA CTC CTC T AAATGAAAAATCTAAAGCA T GGTGCCTAATGAG
6HB-60SE-008	AGGATCCCCGGGTACCGAG T GAT TAA AGT TTA GCA CTC GTC CTA TGT CCA
6HB-control-fold -008	ACTGCCCGAAATTGTCATGGTCATAGCTATCTA TGGATGAAGATGCCTACTTCTAC
6HB-Hinge-3T-control-fold-174	GTAGAAGTAGGCATCTTCATCCAAATGAAAAATCTAAAGCA TTT GGTGCCTAATGAG

Supplementary Table 4.

The DNA used for the substate modification and colloids modification. All DNA are listed from 5' to 3'

Name	Sequence
DBCON-20T-Colloid	/5DBCON/TTTTTTTTTTTTTTTTTTTTCTAGTGCAACAGCAGGTTCAT
Biotin-DNA	Biotin-TTTTTTTTTTTTTTTT TTT TTT GAT CAA AGA GTG CTTAGAG
DBCON-Ref-particle	/5DBCON/ TTTTTTTTTTTTTTTTTTTT CTCTA AGC ACT CTT TGA TC
