# Supplementary material for: A tunable autonomous RNA-fueled micro-engine
Source: Nat Commun. 2026 Feb 25;17:3164. doi: 10.1038/s41467-026-69521-x (PMC13046739; doi:10.1038/s41467-026-69521-x)
Supplement: Supplementary file 2 — Description of Additional Supplementary Files [file 41467_2026_69521_MOESM2_ESM.pdf]

Description of Additional Supplementary Files

Supplementary data 1: caDNAno design file for the six-helix-bundle DNA origami (6HB).

Supplementary data 2: Sequence of specific DNA staple strands for sticky legs of 6HB DNA origami as shown in Supplementary Figure 1. Note: The last three digits in the name represent the position of the staple strand that needs to be replaced.

Supplementary data 3: The specific DNA staple strand sequence is designed for the rigidly connected by six duplexes. Note: The fourth-to-last digit represents the identification number of the 6HB in the tetramer (Supplementary Figure 1). The last three digits in the name represent the position of the staple strand that needs to be replaced.

Supplementary Data 4: The sequences of the 6HB origami staple strands. Note: The last three digits in the name represent the position of the staple strand. All DNA are listed from 5′ to 3′.

Supplementary Data 5: DNA origami scaffold the circular, 7249 bases long, single-stranded DNA scaffold was purchased from Tilibit Nanosystems GmbH, type p7249 (M13mp18) 2 ml at 400 nM. M13mp18, circular single stranded DNA type p7249.

Supplementary movie 1: The RNA-fueled engine is operated in the absence of RNA and RNase H (unfolded control). The left panel shows the time series of the particle's relative position. The middle panel shows a representative two-dimensional trajectory of the particle over time. The right panel shows the raw video of the engine with a dye-labeled colloidal particle (scale bar: 4 µm). The movie is played at 10× real time.

Supplementary movie 2: The RNA-fueled engine is operated in the presence of RNA (100 nM) (folded control). The left panel shows the time series of the particle's relative position. The middle panel shows a representative two-dimensional trajectory of the particle over time. The right panel shows the raw video of the engine with a dye-labeled colloidal particle (scale bar: 4 µm). The movie is played at 10× real time.

Supplementary movie 3: The RNA-fueled engine is operated in the presence of RNA (500 nM) and RNase H (80 nM) at 37 °C. The left panel shows the time series of the particle's relative position. The middle panel shows a representative two-dimensional trajectory of the particle over time. The right panel shows the raw video of the engine with a dye-labeled colloidal particle (scale bar: 4 µm). The movie is played at 10× real time.
