# Supplementary material for: A tunable autonomous RNA-fueled micro-engine
Source: Nat Commun. 2026 Feb 25;17:3164. doi: 10.1038/s41467-026-69521-x (PMC13046739; doi:10.1038/s41467-026-69521-x)
Supplement: Supplementary file 11 — Reporting summary [file 41467_2026_69521_MOESM11_ESM.pdf]

nature portfolio

Corresponding author(s): Kun Wang, Paul M. Chaikin

Last updated by author(s): Jan 7, 2026

Reporting Summary

Nature Portfolio wishes to improve the reproducibility of the work that we publish. This form provides structure for consistency and transparency in reporting. For further information on Nature Portfolio policies, see our Editorial Policies and the Editorial Policy Checklist.

Statistics

For all statistical analyses, confirm that the following items are present in the figure legend, table legend, main text, or Methods section.

n/a	Confirmed	
☐	☒	The exact sample size (*n*) for each experimental group/condition, given as a discrete number and unit of measurement
☐	☒	A statement on whether measurements were taken from distinct samples or whether the same sample was measured repeatedly
☐	☒	The statistical test(s) used AND whether they are one- or two-sided<br>*Only common tests should be described solely by name; describe more complex techniques in the Methods section.*
☐	☒	A description of all covariates tested
☐	☒	A description of any assumptions or corrections, such as tests of normality and adjustment for multiple comparisons
☐	☒	A full description of the statistical parameters including central tendency (e.g. means) or other basic estimates (e.g. regression coefficient) AND variation (e.g. standard deviation) or associated estimates of uncertainty (e.g. confidence intervals)
☒	☐	For null hypothesis testing, the test statistic (e.g. *F*, *t*, *r*) with confidence intervals, effect sizes, degrees of freedom and *P* value noted<br>*Give P values as exact values whenever suitable.*
☒	☐	For Bayesian analysis, information on the choice of priors and Markov chain Monte Carlo settings
☒	☐	For hierarchical and complex designs, identification of the appropriate level for tests and full reporting of outcomes
☒	☐	Estimates of effect sizes (e.g. Cohen's *d*, Pearson's *r*), indicating how they were calculated

Our web collection on statistics for biologists contains articles on many of the points above.

Software and code

Policy information about availability of computer code

Data collection	Time-lapse fluorescence movies were acquired on a Nikon Eclipse Ti inverted microscope with a Plan Apo Lambda 100× oil-immersion objective, using a SPECTRA Light Engine for excitation and an Andor Zyla sCMOS camera controlled by Nikon NIS-Elements software.
Data analysis	Raw movies were preprocessed in ImageJ/Fiji (linear contrast adjustment) before particle tracking. Particle trajectories were extracted and analyzed in Python (v3.13) using the Trackpy package (v0.6.1) together with NumPy and SciPy. The custom Python scripts developed for kinetic analysis, including double-exponential weighted fitting and model simulations, are available in a public GitHub repository (https://github.com/kw2556nyu/A_Tunable_Autonomous_RNA_Fueled_Micro_Engine_Analysis_Code.git) and archived with the permanent identifier DOI: 10.5281/zenodo.18173464. The analysis relies on standard open-source libraries (NumPy, SciPy, Matplotlib) as specified in the repository documentation.

For manuscripts utilizing custom algorithms or software that are central to the research but not yet described in published literature, software must be made available to editors and reviewers. We strongly encourage code deposition in a community repository (e.g. GitHub). See the Nature Portfolio guidelines for submitting code & software for further information.

Data

Policy information about availability of data

All manuscripts must include a data availability statement. This statement should provide the following information, where applicable:

- Accession codes, unique identifiers, or web links for publicly available datasets
- A description of any restrictions on data availability
- For clinical datasets or third party data, please ensure that the statement adheres to our policy

All data supporting the findings of this study are available within the paper, the Supplementary Information, and the accompanying Source Data files. The raw time lapse microscopy movies and full analysis scripts are available from the corresponding author upon reasonable request.

Research involving human participants, their data, or biological material

Policy information about studies with human participants or human data. See also policy information about sex, gender (identity/presentation), and sexual orientation and race, ethnicity and racism.

Reporting on sex and gender	Not applicable; the study did not involve human participants or human-derived material.
Reporting on race, ethnicity, or other socially relevant groupings	Not applicable; the study did not involve human participants or human-derived material.
Population characteristics	Not applicable; the study did not involve human participants or human-derived material.
Recruitment	Not applicable; the study did not involve human participants or human-derived material.
Ethics oversight	Not applicable; the study did not involve human participants or human-derived material.

Note that full information on the approval of the study protocol must also be provided in the manuscript.

Field-specific reporting

Please select the one below that is the best fit for your research. If you are not sure, read the appropriate sections before making your selection.

☒ Life sciences ☐ Behavioural & social sciences ☐ Ecological, evolutionary & environmental sciences

For a reference copy of the document with all sections, see nature.com/documents/nr-reporting-summary-flat.pdf

Life sciences study design

All studies must disclose on these points even when the disclosure is negative.

Sample size	Sample sizes were not predetermined by formal power calculations. Instead, we chose the number of engines (particles) and switching events per condition based on prior single-molecule studies and practical throughput, targeting at least tens of engines and tens to hundreds of dwell-time events per biochemical condition. This provides stable cumulative distribution functions and robust parameter estimates.
Data exclusions	No individual dwell-time events were excluded from the analyses. A small number of movies with obvious technical failure (loss of focus or severe stage drift preventing reliable particle tracking) were discarded.
Replication	All key experiments (engine operation, temperature dependence, RNA and RNase H) were repeated in at least three independently prepared sample chambers and with multiple engines per condition. Qualitative behavior and quantitative trends (τO and τC vs. temperature and concentrations) were reproducible across replicates.
Randomization	Sample allocation was not random, as experimental groups were defined by specific biochemical conditions (e.g., varying RNA or RNase H concentrations) required to test the kinetic model. To control for covariates, all experiments used the same buffer composition, surface functionalization protocols, and temperature control (unless temperature was the variable).
Blinding	Blinding was not relevant to this study. The investigators were not blinded to group allocation during data collection and analysis. Blinding was not performed because the experimental setup required the researchers to actively control specific biochemical conditions (e.g., reagent concentrations).

Reporting for specific materials, systems and methods

We require information from authors about some types of materials, experimental systems and methods used in many studies. Here, indicate whether each material, system or method listed is relevant to your study. If you are not sure if a list item applies to your research, read the appropriate section before selecting a response.

Materials & experimental systems

n/a	Involved in the study
☒	☐ Antibodies
☒	☐ Eukaryotic cell lines
☒	☐ Palaeontology and archaeology
☒	☐ Animals and other organisms
☒	☐ Clinical data
☒	☐ Dual use research of concern
☒	☐ Plants

Methods

n/a	Involved in the study
☒	☐ ChIP-seq
☒	☐ Flow cytometry
☒	☐ MRI-based neuroimaging

Plants

Seed stocks

No plant materials, seed stocks, or novel plant genotypes were used in this study.

Novel plant genotypes

N/A

Authentication

N/A
