# Supplementary figures and images for: A tunable autonomous RNA-fueled micro-engine
Source: Nat Commun. 2026 Feb 25;17:3164. doi: 10.1038/s41467-026-69521-x (PMC13046739; doi:10.1038/s41467-026-69521-x)

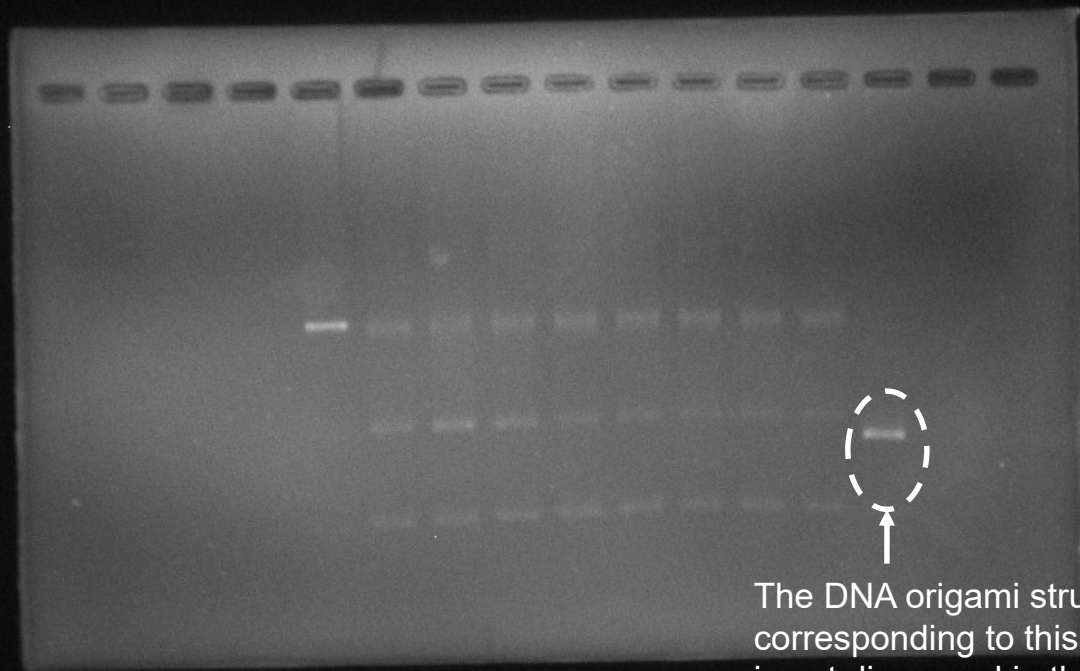

The DNA origami structure corresponding to this band is not discussed in this manuscript.

Supplement: Supplementary file 13 — Source data [file 41467_2026_69521_MOESM13_ESM.zip › Source data/Supplementary Figure 4D.pdf]

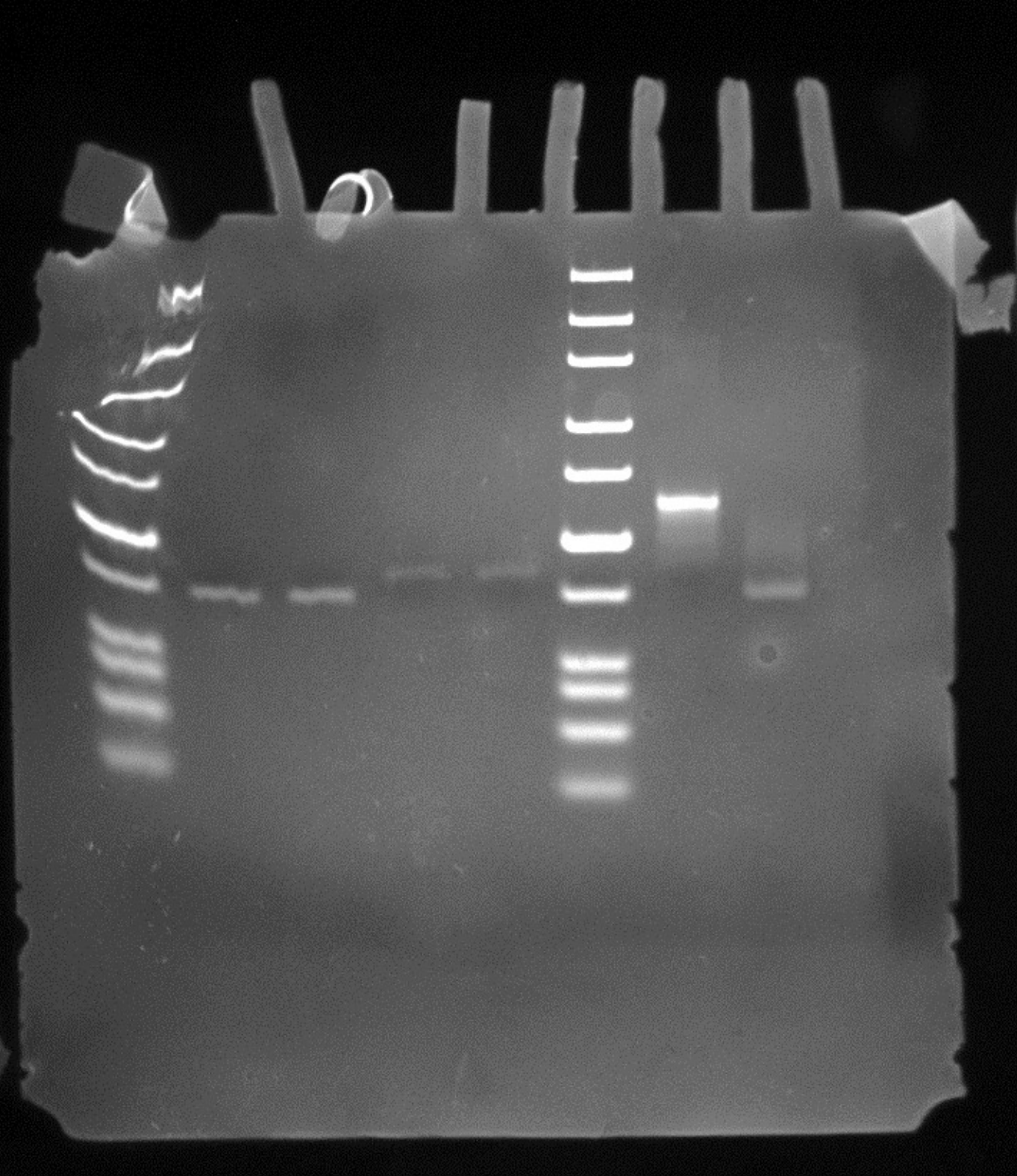

Supplement: Supplementary file 13 — Source data [file 41467_2026_69521_MOESM13_ESM.zip › Source data/Supplementary Figure 3.pdf]

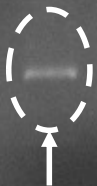

The DNA origami structure corresponding to this band is not discussed in this manuscript.

Supplement: Supplementary file 13 — Source data [file 41467_2026_69521_MOESM13_ESM.zip › Source data/Supplementary Figure 4C.pdf]
